# Detection Dog Survey Detects African Wild Dog Presence and a Shared Marking Site

**DOI:** 10.1002/ece3.71703

**Published:** 2025-07-27

**Authors:** Tim Hofmann, Stijn Verschueren, Tresia Shihepo, Bogdan Cristescu, Nicole Anderson, Nadja le Roux, Shweta Singh, Stephan Neumann, Niko Balkenhol, Laurie Marker, Anne Schmidt‐Küntzel

**Affiliations:** ^1^ Cheetah Conservation Fund Otjiwarongo Namibia; ^2^ Wildlife Sciences, Faculty of Forest Sciences and Forest Ecology University of Goettingen Goettingen Germany; ^3^ Evolutionary Ecology Group, Department of Biology University of Antwerp Antwerp Belgium; ^4^ School of Agriculture and Natural Resource Sciences Namibia University of Science and Technology Windhoek Namibia; ^5^ School of Applied Sciences University of Brighton Brighton UK; ^6^ Kalahari African Wild Dog Conservation Project Swakopmund Namibia; ^7^ College of Science and Engineering Flinders University Adelaide Australia; ^8^ Small Animal Clinic, Institute of Veterinary Medicine University of Goettingen Goettingen Germany

**Keywords:** African wild dog, Kavango‐Zambezi Transfrontier Conservation Area (KAZA TFCA), molecular genetic identification, non‐invasive monitoring, scat detection dog survey, scent‐marking

## Abstract

African wild dog ( *Lycaon pictus* ) populations are difficult to assess effectively and scalable strategies for population monitoring are lacking, often because of low detection rates. Scat detection dogs ( *Canis lupus familiaris* ) have emerged as a suitable tool to detect the presence of wide‐ranging carnivores. In this study, we employed a detection dog to locate African wild dog scat in an unfenced, understudied region of the Kavango‐Zambezi Transfrontier Conservation Area. Over 2 weeks of fieldwork, the detection dog‐team found 21 African wild dog scats within a 2304 km^2^ study area. Six of those scats were detected at a marking site shared by multiple African wild dog individuals, as determined through genetic identification. The marking site discovered by the scat detection dog facilitated the collaring of two African wild dogs in close proximity, the repeat detection of wild dog individuals on camera trap, the collection of additional scat samples, and the highest recording of individuals per site from camera traps (*n* = 5) and genetic verification (*n* = 5). This highlights the value of marking sites for improved long‐term monitoring for this elusive species. To our knowledge, we report the first use of a detection dog to find wild dog scat and discover a shared marking site. Our findings hold promise for the potential of detection dogs to rapidly survey this wide‐ranging, endangered canid.

## Graphical abstract

With the help of our detection dog, we located 21 African wild dog scats, including a shared marking site, during 2 weeks of fieldwork in a non‐protected area in the Namibian section of the Kavango‐Zambezi Transfrontier Conservation Area. The detected marking site enabled further monitoring through repeated scat collection, camera trapping, and facilitated the radiocollaring of two African wild dogs. These findings highlight the potential of detection dogs to improve rapid surveys and long‐term monitoring for this endangered canid.

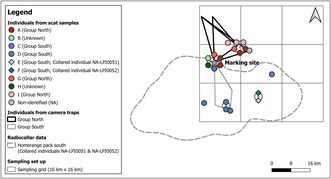

## 1 Introduction

African wild dogs ( *Lycaon pictus* ), hereafter referred to as “wild dogs,” are globally classified as an endangered species with approximately 1400 mature individuals remaining in the wild, making them one of the most threatened large carnivores in Africa (Woodroffe and Sillero‐Zubiri 2020). In Namibia, the majority of the critically endangered wild dog population, estimated at 137–359 adults and yearlings, lives in the north‐east of the country, in unfenced, largely unprotected areas (Hanssen et al. 2022). This increases the likelihood to encounter humans and their livestock, and often results in lethal persecution in response to perceived or actual livestock depredation, which is a major driver for their population decline (Woodroffe and Sillero‐Zubiri 2020). North‐east Namibia is an important region for wild dog conservation, as it has the potential to connect with adjacent areas that provide suitable wild dog habitat (Hofmann et al. 2023) and is part of the Kavango‐Zambezi Transfrontier Conservation Area (KAZA TFCA), which hosts a significant part of the global wild dog population (Woodroffe and Sillero‐Zubiri 2020). However, as in many other countries where they naturally occur, wild dogs in Namibia are studied less intensely than other large carnivores, even though they are more threatened (Strampelli et al. 2022). This disparity is partly due to a combination of ecological and methodological challenges, such as their sociality and wide‐ranging behavior (Woodroffe and Sillero‐Zubiri 2020). Consequently, current density estimates rely largely on resource‐intensive, long‐term monitoring efforts (Strampelli et al. 2022), while other population parameters such as occupancy may be obtained with less effort (Verschueren, Hofmann, Briers‐Louw, et al. 2024).

Searching for indirect signs of presence, such as feces (scat) can facilitate the detection of wide‐ranging and elusive species across diverse environments (MacKay et al. 2008). Scent‐marking with scat is common among carnivore species, with many of them using specific defecation sites known as latrines. These marking sites are characterized by an accumulation of scat from the repeated use by one or several individuals and relate to multiple functions such as resource ownership and information exchange (Buesching and Jordan 2022). Apps et al. (2022) and Claase et al. (2022) recently showed that wild dogs use latrines, some of which are shared by multiple groups and therefore referred to as shared marking sites. Identifying such sites can enhance the detection of wild dogs and thus facilitate efficient population monitoring, for instance, through targeted camera trap placement, which has been successfully applied for other species (Vogt et al. 2014; Verschueren, Hofmann, Briers‐Louw, et al. 2024).

Wild dog latrines are cryptic and finding them may require intensive survey efforts (Claase et al. 2024). Indeed, shared marking sites in wild dogs were reported for the first time in 2022 through direct observations of radio‐collared individuals from a well‐studied subpopulation (Apps et al. 2022). Since capturing and collaring animals can be costly, risky, and time intensive, non‐invasive methods such as scat detection and identification, may be advantageous (Kelly et al. 2012). Wildlife detection dogs ( *Canis lupus familiaris* ) have emerged as an effective, non‐invasive tool to enhance concealed scat sample collection for wide‐ranging species (MacKay et al. 2008), including the rapid identification of cheetah and wolf marking sites (Hofmann et al. 2024; Roda et al. 2022), but thus far they have not been used to detect wild dogs.

Further investigation of wild dogs, particularly in understudied areas such as the Namibian section of the KAZA TFCA, enhances our understanding of the application of detection dogs, supports the generalization of wild dog marking behavior across their range, and thereby aids the management and conservation of the species. We report the use of a detection dog to survey wild dogs and their marking sites in a non‐invasive and time efficient manner. Furthermore, because our study occurred in a communal conservancy, it afforded the opportunity to explore whether wild dogs outside protected areas exhibit marking behavior, which has thus far only been described on protected land (Apps et al. 2022). We verified the findings of the detection dog survey using genetic‐based species and individual identification of wild dogs from scat, and validated our conclusions with camera trap imagery and telemetry data.

## 2 Materials and Methods

### 2.1 Study Area & Survey Set Up

**FIGURE 1:**
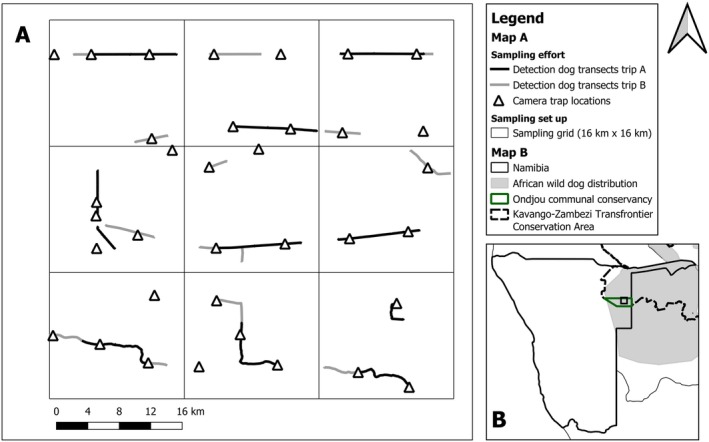
(A) Overview of the study area showing the sampling grid of nine cells measuring 16 km × 16 km each, covering a total of 2304 km^2^; the detection dog transects (Trip A: Black lines, Trip B: Gray lines) and the camera trap locations (black triangles). (B) Location of the study area (black square) in Namibia including the Ondjou communal conservancy (green polygon), the Kavango‐Zambezi Transfrontier Conservation Area (KAZA TFCA; dashed black line), and the distribution of African wild dogs (gray polygon; *source:* IUCN [International Union for Conservation of Nature] 2008. *Lycaon pictus* . The IUCN Red List of Threatened Species. Version 2024‐2).

The Ondjou communal conservancy covers 8729 km^2^ in the Otjozondjupa region of north‐east Namibia, at the southwestern extent of the KAZA TFCA (Figure 1B). The conservancy hosts a diverse carnivore guild, even though wild prey species are scarce due to high hunting pressure exerted by a sparse human population (Verschueren, Hofmann, Kakove, et al. 2024). The vegetation is characterized as a broadleaved tree‐and‐shrub savanna, and the climate is hot and semi‐arid, with a mean annual rainfall of 350–400 mm, concentrated in the wet season between November and March (Atlas of Namibia Team 2022). We conducted our survey during the dry season (July—September 2022), to increase the chances of finding scat. We applied a square grid consisting of nine sampling units each measuring 16 km × 16 km (256 km^2^) resulting in a total study area of 2304 km^2^ (Figure 1A). The size and number of the sampling units was chosen to accommodate the wide ranging behavior of our focal species and to facilitate home range level inferences (Pomilia et al. 2015). The grid location was selected to incorporate a suspected gradient in human pressure deriving from an exclusive wildlife zone to the west and the main human settlement to the east of the grid (Verschueren, Hofmann, Kakove, et al. 2024).

### 2.2 Scat Detection Dog Survey

The “detection dog‐team” consisted of a trained scat detection dog (spayed female Belgian Malinois, 4 years old), a dog handler, and a community game guard as field guide. The detection dog was trained to alert to wild dog scat, following established principles of detection dog training (MacKay et al. 2008). Specifically, the dog was imprinted on the target (wild dog scat) through positive reinforcement, either play or food. The dog was taught to communicate a successful find of a target to the handler by sitting next to it (indication). For imprinting we used 23 wild dog scat samples from nine individuals (7 males and 2 females), sourced from captive wild dogs in rehabilitation at the Cheetah Conservation Fund's (CCF) research and conservation centre in Namibia. After the imprinting stage, scent discrimination was introduced. During this process, negative samples from non‐target species were presented to the dog, which she was taught to ignore by being rewarded for correctly indicating the target species (see Hofmann et al. 2025 for details about dog training and performance metrics).

We sampled on two different occasions with the detection dog‐team: in July (Trip A) and September 2022 (Trip B), corresponding to the beginning and the end of the concurrent camera trap survey. Within each 256 km^2^ sampling unit, the detection dog‐team walked 16 km of individual, non‐repeated transects (10 km during trip A, 6 km during trip B), leading to a total of 144 km of transects along randomly assigned single‐track dirt roads (Figure 1A). Transects were walked during the cooler hours of the day to enhance dog performance, with a maximum of 10 km walked per day. We also opportunistically scouted for wild dog tracks along roads while driving, and in areas where tracks were spotted, we searched for scats with the detection dog. Scats were collected if they were suspected to originate from wild dogs based on either the indication behavior of the detection dog or field identification by the dog handler and the community game guard. The handler, an experienced detection dog handler and ecologist, and the community game guard, with several years of experience in the study area, were both skilled in identifying signs of animal presence. We recorded the GPS location of each scat and classified locations as marking sites if at least three distinct scats were found in close proximity (< 3 m between samples). We brought all scat samples to CCF's Namibia‐based conservation genetics laboratory, where we stored them at −20°C until analysis.

### 2.3 Camera Trap Survey

We deployed camera traps (Browning Strike Force Pro XD, USA) at 35 unbaited locations (see Verschueren, Hofmann, Kakove, et al. 2024 for details about the camera trap survey). In brief, we aimed to place camera traps near the centroid of each grid cell, typically along single‐track dirt roads crossed by wildlife trails, to ensure sampling independence and reduce spatial autocorrelation (Figure 1A). Additional camera traps were placed in areas with increased likelihood of detections, such as accumulations of scats or tracks found during the detection dog survey. To enhance detections and improve individual identification, two cameras were set per location. Cameras operated for 2 months, capturing five‐image bursts per trigger with a 5‐min delay to conserve battery life. We identified individual wild dogs in photographs from their unique coat patterns. We reviewed camera footage to identify the behaviors of sniffing or scent‐marking (defecation, urination, rub rolling, or dragging; Claase et al. 2024). We delineated the spatial coverage of individuals detected at a marking site by creating minimum convex polygons of camera trap locations for any individual detected at more than two locations and a line feature for any individual detected at only two locations using a GIS (QGIS Development Team 2022).

### 2.4 Collaring

On August 2nd 2022, the Kalahari African Wild Dog Conservation Project (KAWDCP) immobilized two adult male wild dogs belonging to the same pack (Deans et al. n.d.), approximately 6.6 km from a marking site, which was located on July 28th 2022 by our detection dog survey. Blood was sampled and a satellite GPS radiocollar (Africa Wildlife Tracking, Pretoria, South Africa) with very‐high‐frequency (VHF) transmitter was attached to each individual to facilitate long‐term monitoring. The collars were programmed to acquire one GPS fix every 4 h and were active for the period corresponding to the detection dog and camera trapping surveys (excluding the 7 days preceding the collaring). We used the collar data to estimate home ranges of the two wild dogs using a 95% kernel density estimate (KDE) with a h_ref_ smoothing factor in the R package “adehabitatHR” (Calenge, Clément 2006). Locational information for both individuals were pooled (*n* = 678) because they were traveling together for the period included in the analysis (60 days).

### 2.5 Genetic Verification

We extracted the scat samples with the QIAamp Fast DNA Stool Mini Kit (QIAGEN) following manufacturer's recommendation, with the exception of the scat amount (only 100 mg for first extraction as per Wong et al. 2025) and the elution volume (only 100 μL elution buffer). If results could not be finalized with one extraction, we performed a second and third extraction (with 200 and 50 mg of scat, respectively). We used up to two PCR amplifications per extraction for species identification and up to six PCR amplifications per extraction for individual identification. We first verified carnivore species with a mitochondrial ATP6 mini‐barcode (primers ATP6‐DF3 and ATP6‐DR1; Haag et al. 2009) through sequence comparison with a carnivore reference sequence database (DRYAD doi: 10.5061/dryad.bcc2fqzmt) using the Geneious Prime 2023.1.1 (https://www.geneious.com) alignment tool with default settings (see Wong et al. 2025 for details about the laboratory work and analysis). When needed, we confirmed species identity of suspected wild dog samples using the allelic profile of known domestic dog microsatellite markers (see Marsden et al. 2012 for multiplex and primer information). We extracted blood samples obtained from the two collared wild dogs (see Collaring section) using the DNeasy Blood and Tissue Kit (Qiagen) following manufacturer's recommendations. We generated individual identities of wild dogs for blood and confirmed scat samples using the same microsatellite markers. We assigned group membership to genetically identified individuals by interpreting data from all methods, the detection dog survey, the resulting genetic analysis, camera trap footage, and the movement data from the radiocollars.

### 2.6 Ethics Statement

The fieldwork was authorized by the Namibian National Commission on Research Science & Technology under Section 21 of the Research Science and Technology Act No. 23 of 2004. The execution of data and sample collection was performed under the research permit number AN2018051701 of the Cheetah Conservation Fund (Namibian‐based Institute RCIV00122018). We conducted the fieldwork with the consent of the executive committee of the Ondjou Conservancy. We adhered to the ethical code of conduct for the use of camera traps in wildlife research (Sharma et al. 2020). The training and handling of the detection dog were endorsed by the animal welfare officer of the University of Goettingen. Immobilization and fitting of GPS collars to the African wild dogs was undertaken by a qualified and experienced wildlife veterinarian registered to practice with the Namibian Veterinary Council.

## 3 Results

**FIGURE 2:**
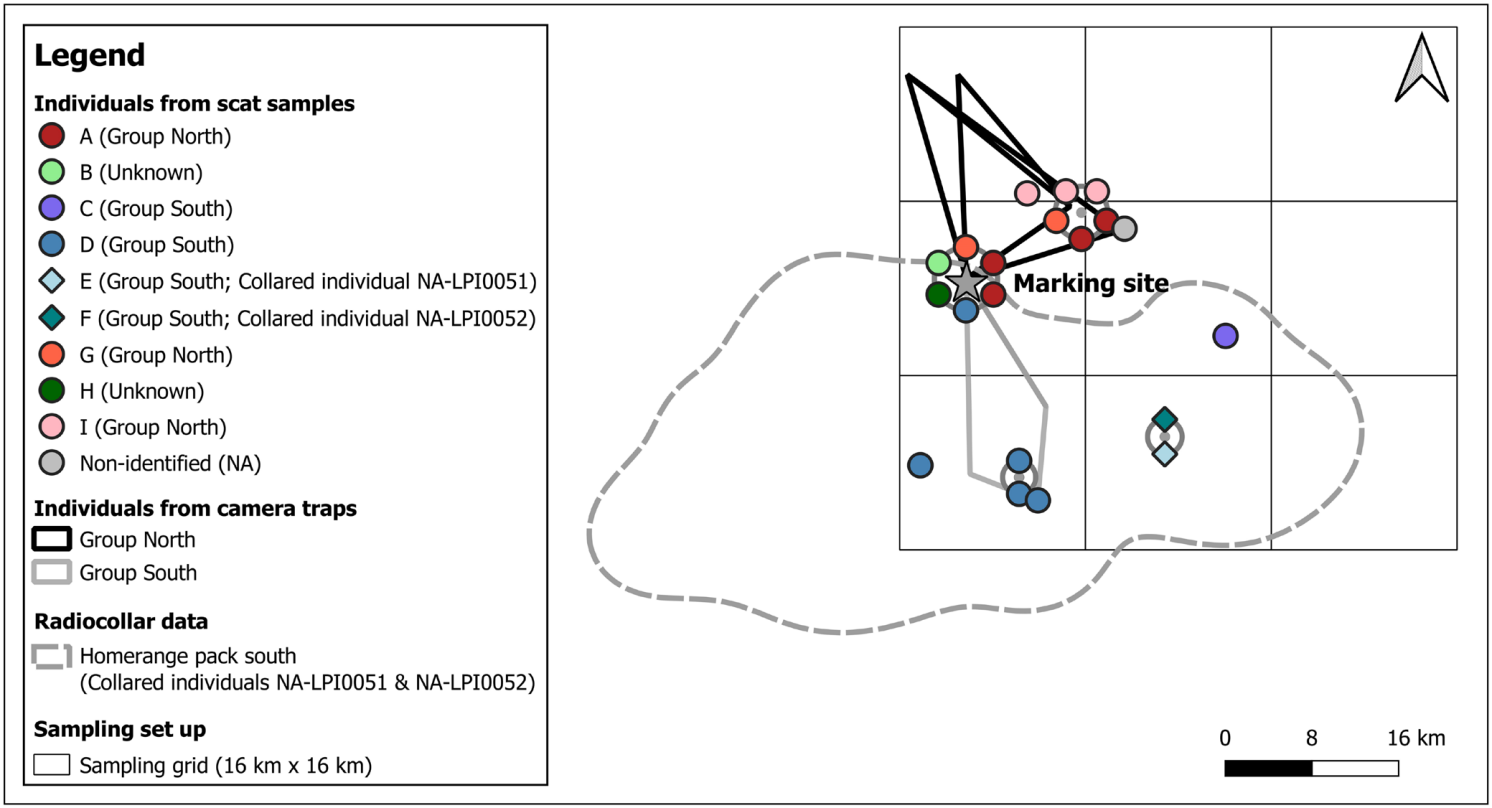
Locations of individual African wild dogs detected using various survey methods in relation to the shared marking site (gray star). Verified African wild dog scat locations are indicated by circles and diamonds, color‐coded according to their genetic individual identification and their inferred group membership (blue shades for south, red shades for north, green shades for only at the marking site, and gray for non‐identified individual) as outlined in Table 1. Overlapping points are displayed using the ‘point displacement’ method in QGIS (QGIS Development Team 2022), resulting in 20 visible points for 21 samples, as individual A was found twice at the marking site (only one sample is shown) and once approximately 100 m south of it (shown as part of the marking site). African wild dogs detected by camera traps at the shared marking site are represented by polygons and lines color‐coded according to their location in relation to the marking site (gray for south [two individuals overlap] and black for north). The dashed gray polygon indicates the 95% kernel density estimate of the merged home ranges for the two collared African wild dogs roaming together around and south of the marking site.

We collected 30 scats that were suspected to originate from wild dogs during road transects (*n* = 25) and opportunistic sample collection (*n* = 5), both executed with the detection dog. Of the 30 samples, we found 23 throughout the study area, while seven were closely together (< 3 m between samples; Figure 2). The latter scats were on and next to a single‐track dirt road, crossed by a wildlife trail, and surrounded by dense vegetation, fulfilling some of the criteria of a marking site according to Apps et al. (2022). Of the samples located at the suspected marking site, three were detected on the first visit and the remaining four on the second visit (63 days later). We found another accumulation of five scats in the north‐east (Figure 2) but they were more widely dispersed and located along a road, suggesting defecation while traveling rather than a marking site. Furthermore, since these were detected during trip B, we did not install camera traps, nor were we able to repeat scat collection, and thus could not further investigate the potential of this being another marking site.

We genetically confirmed six of the seven samples from the marking site as African wild dog using an ATP6 mini‐barcode (*n* = 2) and/or microsatellite marker profiles (*n* = 6) and identified five different individuals (individuals A, B, D, G, and H; Table 1). Of the 23 samples found throughout the study area, we confirmed 15 as wild dog (*n* = 10 with the ATP6 mini‐barcode, *n* = 15 with the microsatellite marker profiles) and identified seven individuals (individuals A, C‐G, I). Three of the seven individuals (individuals A, D, and G) matched individuals found at the marking site. Individual A was found once 100 m south of the marking site (not distinguishable from the marking site on Figure 2) and twice north‐east of the marking site; individual D was found four times south of the marking site, and individual G was found once north‐east of the marking site. In addition, individual I was found only to the north‐east, individuals E and F only to the south‐east, and individual C only to the east of the marking site. Individual E corresponded to the collared individual NA‐LPI0051 and camera trap individual 4, while individual F corresponded to the collared individual NA‐LPI0052, which was not captured on camera.

**TABLE 1:** Genetic individual identification of African wild dogs from scat samples, and the number of scats per individual (#) with their respective location in the study area in relation to the marking site, and whether the samples were found during trip A or B. The inferred group membership was assigned based on genetic, camera trap, and radio telemetry data.

Genetic ID	Scats (#)	Location					Trip	Inferred Group
		Marking site	North‐east	South	South‐east	East		
A	4	+	+	+^a^	−	−	A & B	North
B	1	+	−	−	−	−	A	Unknown
C	1	−	−	−	−	+	A	South
D	6	+	−	+	−	−	B	South
E^b^	1	−	−	−	+	−	B	South
F^b^	1	−	−	−	+	−	B	South
G	2	+	+	−	−	−	B	North
H	1	+	−	−	−	−	B	Unknown
I	3	−	+	−	−	−	B	North
Non‐identified	1	−	+	−	−	−	B	NA

^a^ Only approximately 100 m south of the marking site.

^b^ Individual E corresponds to the collared individual NA‐LPI0051 and camera trap individual 4, while individual F corresponds to the collared individual NA‐LPI0052, which was not captured on camera.

**FIGURE 3:**
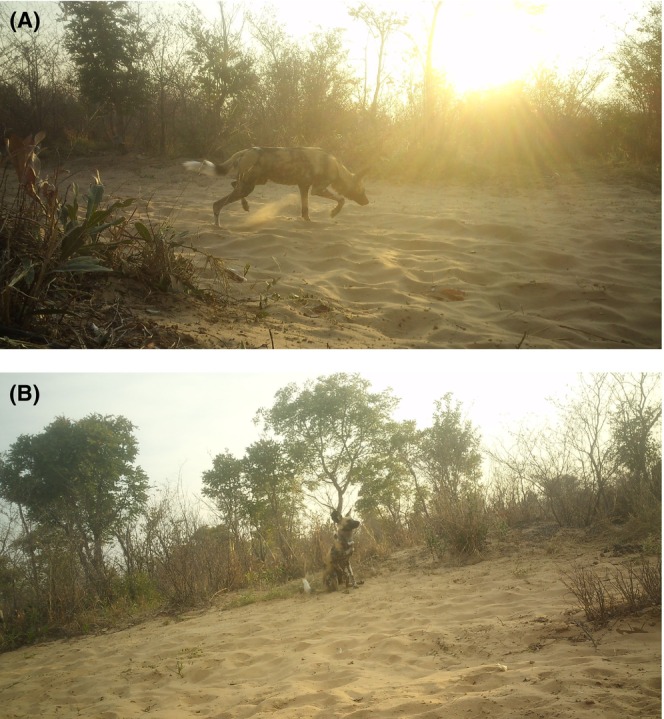
Camera trap photographs of African wild dogs at the shared marking site displaying behaviors of (A) Sniffing and (B) Scent‐marking (dragging) by collared individual NA‐LPI0051. The shared scent‐marking site was detected in the Namibian section of the Kavango‐Zambezi Transfrontier Conservation Area by our scat detection dog complemented with genetic verification and was confirmed with camera trap and radiocollar data.

We recovered 317,758 images over 1963 camera trapping nights and identified 17 individual wild dogs, of which five were seen at the marking site during a total of four events (Table 2). Individual 4, corresponding to the collared wild dog NA‐LPI0051 and genetic individual E, was observed at the marking site, displaying the behaviors of sniffing and scent‐marking in the form of dragging (Figure 3). All five dogs were also detected at other camera trap stations: two individuals were exclusively detected to the south of the marking site, while the remaining three were solely detected to the north and northeast (Figure 2).

**TABLE 2:** Detections of individual African wild dogs on camera trap at the marking site with their respective sex and behavior. The inferred group membership was assigned based on the time between detections (less than 30 min), additional detections in other parts of the study area and genetic, camera trap, and radio telemetry data.

Event	Date	Time	Photographic ID	Sex	Behavior	Inferred Group
**1**	3 Aug 22	7:49 AM	1	F	Sniffing^a^	North
	3 Aug 22	7:49 AM	2	F	Sniffing	North
**2**	3 Aug 22	5:33 PM	3	M	Sniffing	South
	3 Aug 22	5:38 PM	4^b^	M	Scent‐marking (dragging)^c^	South
**3**	15 Aug 22	12:32 AM	5	F	Sniffing	North
**4**	23 Sep 22	6:48 AM	1	F	Sniffing	North

^a^ Event captured in Figure 3A.

^b^ Individual 4 corresponded to the genetic individual E and the collared individual NA‐LPI0051.

^c^ Event captured in Figure 3B.

The pooled movement data from the two individuals that were collared in proximity to the marking site subsequent to its discovery by the detection dog survey resulted in a home range size of 1621 km^2^ extending predominantly south of the marking site, which was located within 1 km of the north‐central home range boundary (Figure 2).

## 4 Discussion

Our detection dog discovered the presence of African wild dogs in large parts of the study area by finding their scat as well as a shared marking site. We were able to derive those findings from only 14 field days, without prior knowledge of the distribution and numbers of wild dogs in the study area. While we used additional survey methods, such as camera trapping and radio telemetry, to confirm the inferred shared nature of the marking site, further detections would have allowed more robust conclusions about wild dog presence and behavior based solely on the findings of the detection dog and genetic verification. More detections by the dog can be achieved with an increased search effort, particularly when executed later in the dry season as scats accumulate.

Genetic verification and camera trap imagery both recorded most individuals at the marking site compared to all other locations surveyed, and the capture of two wild dogs for radiocollar placement occurred in close proximity to the marking site. This highlights the importance of identifying such areas as they can contribute to long‐term population monitoring programs by informing targeted camera trap placement, repeated sample collection for genetic analysis, and collaring operations. It is noteworthy that our study period largely overlapped with the denning season of wild dogs in similar geographic regions (McNutt et al. 2019), during which home ranges are smaller and marking sites visited less frequently (Claase et al. 2022). Therefore, even though denning was not directly observed in this study, it can be assumed that the detection of individuals through genetic sampling and camera trapping at marking sites may be even higher outside of the denning season.

We inferred the existence of the marking site based on field observations at the time of its discovery by our detection dog. Genetic individual identification of the scat samples revealed that the site was used by multiple wild dogs and possibly shared between wild dog groups, classifying it as a shared marking site. The existence of multiple groups was postulated based on the spatial separation observed between individuals in the north and south of the study area, which intersected at the marking site. We further verified our findings from the scat detection dog survey with the more frequently used methods of camera trapping and radio telemetry. Indeed, the camera trap data aligned with the proposed spatial separation because individuals photographed in the north were not photographed south of the marking site and vice versa. The collar data outlined the home range of a group in the south, encompassing the individuals detected from the genetic and camera trap data. Together, these findings strongly suggest two distinct wild dog groups or, at least confirm that not all individuals are part of a single pack, and thus the characterization of the marking site as shared marking site.

The movement data indicates that the shared marking site is near the southern group's home range boundary, which extends approximately 1 km north of the shared marking site. The distribution of scat from genetic individual A suggests that individuals from the north may also venture slightly south of the shared marking site. Based on these data, we can cautiously infer slightly overlapping home ranges, which include the shared marking site at their intersection. As such, our findings largely align with the observations of Apps et al. (2022) that wild dogs use latrines; that the latrines are used by more than one group of individuals on multiple occasions; that latrines are located near home range boundaries; and that latrines are located on or near a road, at or near junctions. In our case, the junction consisted of a wildlife trail crossing the road. Therefore, our findings further support the existence of shared marking sites in this species, including outside protected areas, and suggest their occurrence even in systems with potentially lower wild dog density and higher human pressure than previously described (Woodroffe and Sillero‐Zubiri 2020; Apps et al. 2022; Hanssen et al. 2022; Verschueren, Hofmann, Kakove, et al. 2024).

The large home ranges of wild dogs make inter‐pack communication both challenging and energetically costly (Claase et al. 2022) and the use of marking sites may be an effective territoriality strategy, as demonstrated by other wide‐ranging African species (Rafiq et al. 2020; Vitale et al. 2020; Caro 1994). Further investigating the scent‐marking behavior of wild dogs holds significant value beyond population monitoring, and in particular for human‐wildlife conflict mitigation. For example, through exploring the application of artificial chemical signals, currently under investigation in the “Bio Boundary Project” (Botswana Predator Conservation Trust 2023), to effectively steer the movement of wild dogs away from conflict hotspots (Jackson et al. 2012). Additionally, as demonstrated in this investigation, the discovery of a shared marking site facilitated the capture and collaring of two wild dogs, which are now being monitored for human‐wild dog conflict mitigation (KAWDCP, unpublished data).

We demonstrated that detection dogs can rapidly confirm wild dog presence and locate shared marking sites without previous knowledge of wild dog presence in the study area. This knowledge can be instrumental for effective research and conservation efforts and gives insights into the behavioral ecology of the study species, especially when combined with other non‐invasive methods such as genetic verification and camera trapping. This approach is also applicable to other wide‐ranging carnivores, highlighting the value of detection dogs as a survey methodology.

## Author Contributions

**Tim Hofmann:** conceptualization (lead), data curation (equal), funding acquisition (equal), investigation (lead), visualization (lead), writing – original draft (lead), writing – review and editing (lead). **Stijn Verschueren:** conceptualization (equal), data curation (equal), funding acquisition (equal), investigation (equal), writing – review and editing (equal). **Tresia Shihepo:** data curation (equal), investigation (equal), validation (supporting), writing – review and editing (equal). **Bogdan Cristescu:** conceptualization (equal), project administration (equal), supervision (equal), writing – review and editing (equal). **Nicole Anderson:** data curation (equal), writing – review and editing (equal). **Nadja le Roux:** investigation (equal), writing – review and editing (equal). **Shweta Singh:** data curation (supporting), investigation (supporting), writing – review and editing (equal). **Stephan Neumann:** supervision (equal), writing – review and editing (equal). **Niko Balkenhol:** funding acquisition (equal), supervision (equal), writing – review and editing (equal). **Laurie Marker:** conceptualization (equal), funding acquisition (lead), project administration (equal), supervision (equal), writing – review and editing (equal). **Anne Schmidt‐Küntzel:** conceptualization (equal), data curation (equal), funding aquisition (supporting), project administration (equal), supervision (equal), validation (lead), writing – original draft (supporting), writing – review and editing (lead).

## Conflicts of Interest

The authors declare no conflicts of interest.

## Data Availability

The authors elect to not share data due to its sensitive nature, which could risk poaching, collection, or harassment of the study animals.
